# Attribution of Responsibility Between Agents in a Causal Chain

**DOI:** 10.1111/cogs.70280

**Published:** 2026-11-10

**Authors:** Vanessa Cheung, Mengxuan Helen Qiao, Teneille Brown, David Lagnado

**Affiliations:** ^1^ Department of Experimental Psychology University College London; ^2^ S.J. Quinney College of Law, The University of Utah

**Keywords:** Moral judgment, Moral decision‐making, Blame attribution, Causal responsibility, Causal chain, Proximate cause, Superseding cause

## Abstract

In a “hired gun” killing, who do ordinary people consider more responsible—the person who instigated or the person who executed the killing? We explored the attribution of causal and moral responsibility in a causal chain, where an agent A instructs an intermediate agent B to execute some action harming victim V. Across four studies (two preregistered), we consistently found that participants attributed more causality and blame to the intermediate agent B. In Study 2, we manipulated proximity by adding a third, more direct contributing cause that occurred after A and B's involvement. Participants judged both agents to be less causal, blameworthy, and deserving of punishment when they were less proximal to the outcome, but still rated B more responsible than A in all conditions. In Study 3, we manipulated the intentionality of both agents, and in Study 4, we jointly manipulated intentionality and proximity. We showed that higher intentionality largely drove harsher judgments for each agent, and A's high intent also reduced blame for B. We also found that intentionality had a larger effect on judgments than proximity. We discuss explanations for our findings and their implications for moral and legal decision‐making.

## 1 Introduction

In February 2022, Jennifer Faith, who had instructed her boyfriend Darrin Lopez to kill her husband, pleaded guilty to a murder‐for‐hire charge. This offense carries a potential death sentence in the state of Texas.^1^ Darrin Lopez, the “hired gun,” was later also convicted of murder.^2^ There are a number of fact‐specific considerations for how the two defendants are sentenced in cases like these. However, we are interested in understanding folk perceptions of causal responsibility when an individual commits harm “by proxy.” Do ordinary people consider Faith and Lopez to be equally responsible and blameworthy for the victim's death? Do the two deserve equal punishment? These questions address how people attribute responsibility to multiple agents when they have each contributed differently to an injury.

One key difficulty in evaluating causality in such situations is that there are many factors that can combine in various ways to cause an outcome. The causal structures of realistic scenarios are often complex and multifactorial (DeScioli & Kurzban, 2013). People also attribute responsibility differently depending on how these causal factors interact (Gerstenberg & Lagnado, 2010), and causal judgments are often graded instead of binary. In cases that feature multiple agents, it is often difficult to determine which agent is more responsible for the outcome, and by how much.

There are at least three causal structures in which two agents A and B might be responsible for a single outcome: (1) both agents independently contribute to cause an outcome; (2) the actions of A cause the actions of B, which directly cause the outcome, and (3) the group, of which A and B are both part of, jointly causes the outcome (Kaiserman, 2021). While many studies have explored how people attribute responsibility in (1) (e.g., Gerstenberg & Lagnado, 2010), fewer have explored situations such as (2) where B's actions depend on A in a causal chain.

This is a case of joint causation, where both agents are necessary for the outcome and mutually interdependent. While A did not directly cause the outcome, it would not have occurred but for A's instigation; on the other hand, while B physically caused the outcome to occur, B would not have done so in the first place had it not been for A's initial instigation. Consider the case described earlier: Do people consider Faith, who ordered the killing, to be more responsible, as none of the subsequent events would have happened *but for* her setting things in motion? Or do they consider Lopez more responsible because he *directly and physically* caused the victim's death? Legally, the person who hires a killing (A) is generally treated as at least as culpable, and often more culpable, than the person who physically carries it out (B). Thus, the criminal law in the United States and the United Kingdom does not rank the “trigger‐puller” as more blameworthy simply because they performed the act.

### 1.1 Joint responsibility in a causal chain

Harms resulting from the behavior of multiple agents are causally complex, especially in moral or legal contexts. Imagine a scenario where two agents, A and B, jointly cause an outcome: A throws a lighted cigarette into a shrub which catches fire, and the fire is exacerbated by B pouring petrol on the flames. Hart and Honoré (1985) argue that people are more likely to attribute causality to the proximal cause (B) since it is an intentional human action that intervened in the causal chain, regardless of whether the distal cause (A) is a human action or a physical cause. Conversely, if the proximal cause was a physical cause (e.g., instead of B pouring petrol, the wind had exacerbated the flames), then people are more likely to attribute causality to A's actions. Other studies have supported this theory, finding that people prioritize voluntary actions as explanations over physical causes (Hilton, McClure, & Sutton, 2010; McClure, Hilton, & Sutton, 2007).

However, note that each agent acted independently in the above example: B did not act in concert with A, nor did A's actions bring about B's actions (McClure et al., 2007). As such, this chain of events has been described as a “collider” with a temporal delay between causes rather than a causal chain (see Fig. 1). In such temporal chains, there is evidence that people tend to attribute more causality to the proximal, rather than distal, cause (Lagnado & Channon, 2008; Reuter, Kirfel, Van Riel, & Barlassina, 2014).

**Fig. 1:**
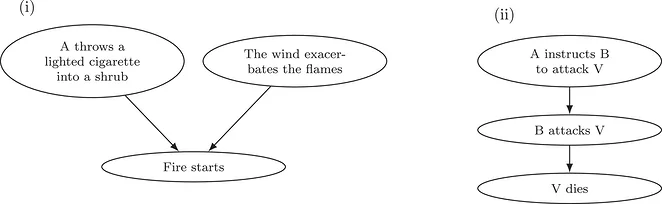
Comparison of a temporal versus causal chain. The left model (i) is a common‐effect model (i.e., a collider), where two causes jointly and independently contribute to the outcome, but at different points in time. This is not a causal chain, as A did not cause the wind's presence. The right model (ii) is a causal chain model, where A causes an intermediate event B contributing to the outcome.

In a causal chain, the concept of “proximity” depends on the number of causes between an action and outcome; the fewer mediating causes, the more proximal the cause (Sloman, Fernbach, & Ewing, 2009). Of the studies that investigate true *causal* chains rather than collider temporal chains, they have either used more abstract vignettes that do not involve human agents (Engelmann & Kirfel, 2025), or involved situations where B acts unknowingly or under some situational constraint brought about by A (Fincham & Shultz, 1981; Phillips & Shaw, 2015). To our knowledge, no research has focused on causal and moral judgments in situations where A and B act *in concert* with each other, but take on different roles and at different times. We, therefore, aim to investigate how people make causal and moral judgments for agents who operate differently in relation to proximity, foreseeability, and intent. The example we use is the case of an instigator instructing someone to execute a criminal act.

### 1.2 Theories of causation

How might different theories of causation help illuminate the psychological factors that shape causal attribution in a causal chain? According to process theories, such as the force dynamics theory (Wolff & Thorstad, 2017), causality involves transmission of a physical quantity along a causal pathway, and an interaction between an event and outcome (such as with dominoes falling; Dowe, 1992). On this view, the proximal cause is more directly and temporally close to the outcome than the distal cause (Hart & Honoré, 1985). This notion of physical directness may influence psychological judgments of causation as well as moral evaluations: people tend to draw stronger causal inferences when an action involves personal force or a physical exchange of forces (Cushman, Young, & Hauser, 2006; Greene et al., 2009), and people also tend to judge the proximal cause to be less morally permissible (Engelmann & Waldmann, 2022).

According to dependency theories, an event is only a cause if it made a difference to the outcome, for example, if it raises the probability that the event would occur (Davis, 1988). One way to evaluate this is through counterfactual simulation (Gerstenberg, Goodman, Lagnado, & Tenenbaum, 2021; Quillien & Lucas, 2023): considering alternative possibilities of how the events could have unfolded. Factors such as the proximity of each agent and the outcome, as well as the agents' mental states, could influence what counterfactuals people imagine and, therefore, their judgments of cause and blame. In the causal chain example illustrated above, where A tells B to harm V, leading to V's death, one might evaluate how the outcome would change if the candidate cause were changed, but not removed (Gerstenberg et al., 2021; Lewis, 1973). Such judgments are ambiguous in a causal chain, as changes to either agent could plausibly affect the outcome. Alternatively, one could ask whether the outcome would still have occurred if a candidate cause were removed entirely. Here, removing A would very likely have prevented the outcome (as B would otherwise lack the information or intention to attack V), whereas removing B would not (A could have hired somebody else or acted himself). This suggests that A may be seen as a more robust or necessary cause than B (cf. Wu & Gerstenberg, 2024). It, therefore, remains an open question how these evaluations influence people's moral and causal judgments of two agents in a causal chain.

### 1.3 Causal chains in the law

To be found guilty of a crime, a defendant's actions must have *caused* the alleged harm. However, in the case of “hired gun” killings, each party's relative causal responsibility is often difficult to determine. How, then, do courts handle scenarios where subsequent acts or events “intervene” between an original actor's criminal conduct and the ultimate injury? When is the second action sufficient to break the causal chain and relieve the initial actor of liability?

The answer is that it depends. For an intervening cause (or *novus actus interveniens*) to relieve the original actor of legal liability, the key question, in both the United States and the United Kingdom, is whether the second‐in‐time “individual's conduct is so unusual, extraordinary, or bizarre” that it breaks the causal chain (*People v. Cervantes*, 2001). These adjectives address the issue of foreseeability, which is a key concept judges use to decide whether an intervening act breaks the causal chain. If the second act is deemed unforeseeable, it is considered a superseding cause, relieving the original actor of liability (e.g., *Regina v. Halliday*, 1889). However, foreseeability is not the sole factor when assessing causation and responsibility. An intervening act that is voluntary, independent, criminal, remote, or indirect can also break the chain of causation. Depending on the facts of the case, some of these variables may correlate with foreseeability, and some may not.

When the different factors relevant to assessing an intervening act conflict with each other, multiple competing tests can be employed, and courts tend to apply them inconsistently (see *People v. Stan XuHui Li*, 2019; *State v. Wade*, 2008; *State v. Wassil*, 1995 for examples of different tests for intervening causation leading to different outcomes).

Foreseeability operates at different levels of abstraction. Unfortunately, there is no judicial agreement on how specific or general its target should be. Defendants may be found guilty if they should have foreseen the general type of harm, even if they could not have foreseen the specific pathway by which it occurred or the specific way that the risk manifested (Abrams, 2025; Johnson, 2025). Thus, judges may inadvertently impact the causation analysis by choosing to be more or less precise in describing what exactly the defendant should have foreseen.

Let us reconsider the “hired gun” murder example. In a straightforward case, such as the one described in the Introduction, the victim's death is a foreseeable outcome to the instigator. But imagine a scenario where A only instructs B to *attack* V, not necessarily intending V's death (as in Fig. 1). In such cases, can A serve as a legal cause of V's death? Can B? When does the action of B relieve A of criminal liability?

Divergent responses to this type of case reveal conflicting moral intuitions and the importance of case‐specific facts, which together may be driving inconsistencies in the applicable legal tests. To analyze which factors are driving the moral and legal assessments, it is important to investigate folk perceptions of causality. This is because the question of causation in the court of law “must be decided as a properly instructed and reasonable jury would decide it,” rather than using a “logical or scientific theory of causation” (*Stapley v. Gypsum Mines*, 1953). But if the various factors are not properly disambiguated, we cannot say whether it is proximity, foreseeability, intentionality, or something else that is motivating these judgments.

### 1.4 Intentionality and foreseeability in causal chains

In addition to foreseeability, the intentionality of the actor is likewise crucial for determining whether an agent should be held accountable for their actions (e.g., Critcher, Helzer, & Tannenbaum, 2020; Malle, Guglielmo, & Monroe, 2014; Shaver, 1985). Alicke (2000) argues that intentional actions include two components: intention of action and intention of outcome. The former refers to whether the agent behaved knowingly, purposefully, and without external constraint; whereas the latter refers to whether the agent caused outcomes that they desired or predicted (Alicke, Mandel, Hilton, Gerstenberg, & Lagnado, 2015). Indeed, people tend to attribute more blame to agents who intend and plan to harm others compared to those who cause accidental or uncontrollable harm, and also attribute more blame for actions with highly foreseeable outcomes (Ames & Fiske, 2013; Lagnado & Channon, 2008). Agents, therefore, can be held differentially accountable for joint causation of an outcome if there is variation in the intentionality of their actions and the degree to which they could foresee the outcome.

In causal chains where two agents act independently, people's causal judgments are not typically influenced by the distal agent's intentions (Brickman, Ryan, & Wortman, 1975; Hilton et al., 2010; McClure et al., 2007). Lagnado and Channon (2008) directly manipulated the intentionality of both (independent) agents and found an overall effect, whereby intentional agents are judged as more causal. However, it remains unclear how intentionality affects responsibility attribution for multiple agents in a causal chain of events where they act *in concert* with each other. In the “hired gun” scenario mentioned earlier, B would know or be able to reasonably infer A's motives. However, B's motives are more opaque to A. Would the causal chain be broken if B develops their own motive of wanting V dead, which leads to an outcome that A did not foresee? Or would laypeople, who could one day serve on juries, consider the outcome to be reasonably foreseeable to A, who set the events in motion?

Another critical question arises as to whether a subsequent act by a third party (e.g., the victim themselves) may break the causal chain between the defendants’ act and the outcome. Previously, we discussed how in the law, such interruptions to the causal chain can relieve responsibility of the distal agent(s). But do laypeople make judgments of responsibility consistent with these legal assumptions? We aim to answer these questions in this article, and build on existing work on how folk and legal ascriptions of causation are similar and where they differ (e.g., Güver & Kneer, 2023; Knobe & Shapiro, 2021).

### 1.5 Overview of studies

Because of the multiplicity of potentially conflicting factors present in these cases, we designed experiments investigating laypeople's moral and causal judgments of two agents who caused harm in a causal chain: working in concert with each other, but contributing in different ways and at different times. Participants read vignettes based on real criminal cases in which two defendants, an instigator (A) and an executor (B) of the criminal act, contribute jointly to harm a victim (V). In all the scenarios that we use, A does not directly harm V but instructs B to do so. We manipulated the extent to which the agents’ conduct was proximate (i.e., temporally close and direct) and intentional. Across four studies, we investigated how people attribute causality, blame, and punishment to two agents in a causal chain. In Study 1, we investigated the effect of an agent's role in the causal chain on causal and moral judgments (i.e., judgments of causality, blame, and punishment) using a short scenario based on a criminal case. In subsequent studies, we explored the role of proximity and intent. In Studies 2 and 3, we varied the proximity of the two agents’ contributions to the outcome (i.e., how much B directly, and in a temporally closer manner, caused the outcome) and whether each agent intended for the outcome to occur to explore how these factors influence laypeople's causal and moral judgments. In Study 4, we investigated the relationship between proximity and intentionality. All materials, data, and analyses are available at the OSF repository (https://osf.io/bg5xz/).

## 2 Study 1

First, we investigated whether people would assign the same or different levels of causal responsibility, blame, and punishment depending on an agent's role in a causal chain.

### 2.1 Method

#### 2.1.1 Participants

The study was approved by the UCL Psychology Ethics Committee (EP/2018/005), and we obtained informed consent from all participants. We conducted a power analysis using G*Power (Erdfelder, Faul, & Buchner, 1996) for a two‐tailed paired samples *t*‐test. To achieve 80% power for detecting an effect size of *d_rm_* = 0.39 (indicated by results of a pilot study) at an alpha‐level of 0.05, 54 participants were required.

We recruited 90 UK participants from Prolific (*M*_age_ = 38.5 years, *SD*_age_ = 13.2 years, 45 male, 45 female; paid £0.70). Participants were prescreened to only include those who were fluent in English, had an approval rate of *≥* 95% on the platform, and had previously participated in at least five studies. We had planned to exclude participants who gave a nonsensical or blank response in the open response question, which also served as a comprehension check; all participants passed this check, and none were excluded.

#### 2.1.2 Design, materials, and procedure

The study used a within‐subjects design. We measured participants’ judgments of causal responsibility, blame, and punishment for the two agents (A and B). They were shown two scales for each measure on a scale of 0–100, one for giving a response for A, and the other for B.

We presented an original vignette in an online experiment on Qualtrics. This described a legal case, loosely based on *R v. Rook* (1993), where A (“Adam”) instructed an intermediate agent B (“Ben”) to attack V (“Veronica”), which led to her death. Both parties admitted to their actions to reduce ambiguity about credibility. The vignette (“Killing”) is shown below:

*Adam Smith and Ben Parker are on trial for the death of Veronica Brown. In the trial, it was stated that Ben caused severe injuries to Veronica in her home on Thursday November 10 2022. According to the forensic report, Veronica died soon from the injuries, and her body was found in the early hours of the following day*.

*Evidence showed that Adam used an online platform to initiate contact with Ben prior to the victim's death. On Monday 7 November (three days before the victim's death), the pair had met in person. During their conversation, Adam provided specific instructions for the attack, and told Ben to cover it up so it would look like an accident*.

*At the trial, Ben admitted to attacking Veronica, but stated that he would not have done so if Adam had not instructed him to. Adam insisted that despite his involvement, the killing was ultimately executed by Ben and that Ben's actions were the main cause of Veronica's death*.

Participants then gave their responses to the questions “*To what extent was each defendant the cause / blameworthy / deserving of punishment for the victim's death?”*. Responses to the first two questions were made on a scale of 0 (Not at all) to 100 (Completely), and for punishment on a scale of 0 (No punishment) to 100 (Maximum punishment [i.e., life imprisonment]). The order of the agents’ names in the questions was counterbalanced between participants.

To ensure robustness of our findings, participants made their judgments using two different kinds of measures. Each participant gave judgments twice with two sets of instructions presented in counterbalanced order. In one version of the questions, participants could freely give ratings to each defendant (i.e., free allocation; “*You may use any number on the scale that accurately reflects your judgments for each defendant, i.e., there are no restrictions.”*). In the other version, participants were told that for each measure, their responses for the two defendants could only total 100 (i.e., fixed sum; “*The judgments you give for the two defendants must add up to 100 for each question.”*). The purpose was to control for potential effects driven by different instructions, and to see whether causal attribution is conceived of as a zero‐sum phenomenon (Kaiserman, 2021).

Lastly, we asked three open‐response questions related to their reasoning process, partially as a comprehension check to ensure that participants read and understood the scenario (data available on OSF).

#### 2.1.3 Data analysis

First, we adjusted the free allocation ratings to be comparable with the fixed sum ratings by rescaling the former to show the proportion of scores given to each agent. For each participant, we then averaged across the fixed sum and rescaled free allocation ratings to obtain one rating for each agent. We conducted paired *t*‐tests to compare mean judgments for agents A and B separately for each judgment measure (cause, blame, and punishment).

### 2.2 Results

As shown in Fig. 2, overall, participants were more severe in their moral judgments for B compared to A. Participants judged B (*M* = 58.4, 95% CI [55.6, 61.1]) to be more of a cause than A (*M* = 41.6, 95% CI [38.9, 44.4]), *t*(89) = 6.06, *p* < .001, *d_rm_* = 1.27. They also judged B (*M* = 54.2, 95% CI [52.2, 56.1]) to be more blameworthy than A (*M* = 45.8, 95% CI [43.9, 47.8]), *t*(89) = 4.27, *p < *.001, *d_rm_* = 0.89. Further, they judged B (*M* = 54.4, 95% CI [52.5, 56.3]) to deserve more severe punishment than A (*M* = 45.6, 95% CI [43.7, 47.5]), *t*(89) = 4.67, *p < *.001, *d_rm_* = 0.98. We found similar results in an analysis of only free allocation responses (see Fig. A1 in the Supplementary Appendix).

**Fig. 2:**
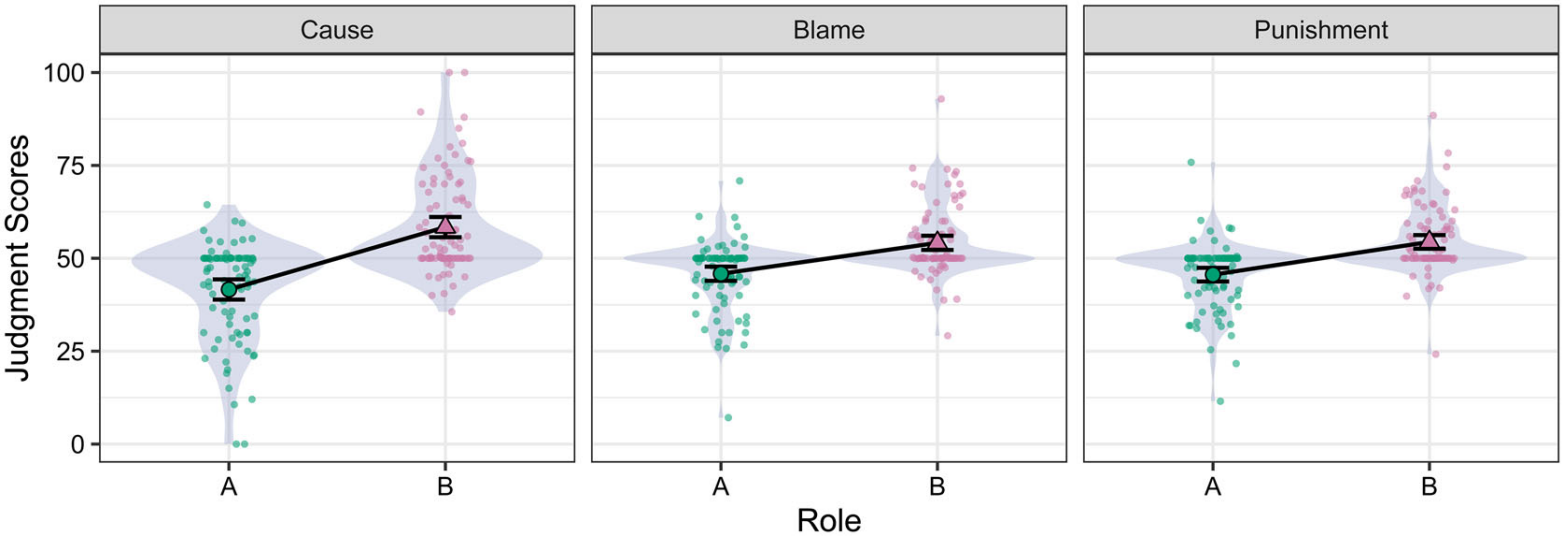
Moral and causal judgments for each agent in Study 1. Jitters show individual participant responses. Error bars indicate 95% CI.

### 2.3 Discussion

Participants tended to rate the executor (B) as significantly more causal, more blameworthy, and more deserving of severe punishment than the instigator (A). This is in line with previous findings showing that people tend to consider the proximal agent to be more of a cause (e.g., Engelmann & Waldmann, 2022; Lagnado & Channon, 2008; Reuter et al., 2014), and judge causing harm in a manner that more directly impacts the victim to be more morally wrong (Greene et al., 2009). Considering that the intentions of both agents are vague in this scenario, and based on the extant psychological literature, there are two key explanations as to why participants tended to attribute more responsibility to B: (1) B directly and physically produced the outcome in a temporally closer manner (Greene et al., 2009; Lagnado & Gerstenberg, 2017) and (2) assumptions about intent and foreseeability. In other words, it may be because B's actions directly produced the outcome, or because participants inferred that B developed his own motives for wanting to kill V (A might have merely wanted to *attack* V, and not necessarily for V to die). We explore these two explanations in Studies 2 and 3.

## 3 Study 2

Study 1 revealed that participants judged the proximal agent (the executor, B) to be more causal, more blameworthy, and more deserving of severe punishment than the distal agent (the instigator, A). Participants may have assigned more causality and blame to B over A because B was more proximal: his actions were closer in time to the outcome, and more directly caused it. Here, we tested this prediction by manipulating the directness of B's actions by adding in a third contributing factor (V's own behavior), which became the last in time and the direct cause of the final outcome (V's death). We investigated people's judgments of the two agents when B's actions were no longer the most proximal to the final outcome.

This study was a preregistered replication of Study 2 presented in Cheung, Qiao, and Lagnado (2024). We also extended the original study by including two more vignettes that follow the same general causal chain structure of the “Killing” vignette. However, here we varied the severity in the final outcome, such as injury instead of death, to improve the generalizability of our previous findings. We preregistered the study at https://osf.io/xrqaw/.

### 3.1 Method

#### 3.1.1 Participants

The study was approved by the UCL Psychology Ethics Committee (EP/2018/005), and we obtained informed consent from all participants. We conducted an a priori power analysis based on the results of a previous study. Cheung et al. (2024) found significant main effects of proximity (*η_p_* ^2^ = 0.04) and role (*η_p_* ^2^ = 0.08) on causal judgments. We powered the study to be sufficient for detecting the main effects of proximity and role. To achieve 80% power for detecting an effect size of *f* = 0.20 at an alpha‐level of 0.05, 216 participants were required.

We recruited 270 UK participants from Prolific using the same exclusion criteria as in Study 1 (paid £0.75 for the 5‐min study). As preregistered, we excluded 14 participants for failing the attention check, which asked what they just read in the vignette. The final sample size was *N* = 256 (*M*_age_ = 42.4 years, *SD*_age_ = 14.0 years, 127 male, 129 female).

#### 3.1.2 Design, materials, and procedure

This study used a 2 (between‐subjects; Proximity) *×* 2 (within‐subjects; Role) *×* 3 (between‐subjects; Vignette) design. Participants were randomized into two groups (low vs. high proximity) and saw one of three possible vignettes. For example, in the “Killing” vignette, in the high proximity condition, participants saw the same vignette as in Study 1 where B directly causes the final outcome. In the low proximity condition, participants saw a similar vignette except V survives the attack. V's leg is permanently damaged by the attack, and she later dies from falling down a flight of stairs due to this injury. We included two other vignettes, “Lake” and “Trespasser” (the latter was loosely based on *Republic of Hawaii v. Oishi*, 1895). Full materials for all vignettes are available on OSF (https://osf.io/bg5xz/).

We used the same measures and procedure as Study 1, except with only free allocation instructions so as not to explicitly encourage comparison between the two agents. We asked participants two multiple‐choice attention check questions after they made all judgments about what happened in the passage (e.g., which agent physically and directly carried out the attack). Participants also rated the causal responsibility and blameworthiness of the victim (V). They also answered the question “*How foreseeable do you think V's death was to each person?”* on a scale of 0–100. We included this question in Study 2 because, given the addition of the victim's action later in the causal chain, participants may now judge the final outcome to be less foreseeable to the agents. This may be a possible explanation as to why they reduce judgments of responsibility for the agents.

#### 3.1.3 Data analysis

We conducted 2 (Proximity) *×* 2 (Role) *×* 3 (Vignette) mixed ANOVAs for judgments of cause, blame, and punishment separately using afex (Singmann et al., 2022), and pairwise comparisons using emmeans (Lenth, 2022) with Tukey‐adjusted *p*‐values. As we did not sufficiently power the study to test for effects within each vignette, the preregistered main analyses focused on testing the main effects of proximity and role averaged across all vignettes.

### 3.2 Results

#### 3.2.1 Main analysis

As shown in Fig. 3, on average, participants judged B to be more of a cause than A, *F* (1, 250) = 110.81, *p < *.001, η^2^_*p*_ = 0.31, more blameworthy than A, *F* (1, 250) = 76.21, *p < *.001, *η_p_* ^2^ = 0.23, and more deserving of severe punishment than A, *F* (1, 250) = 90.25, *p < *.001, *η_p_* ^2^ = 0.27. They also judged that the final outcome was more foreseeable for B than for A, *F* (1, 250) = 43.27, *p < *.001, *η_p_* ^2^ = 0.15.

**Fig. 3:**
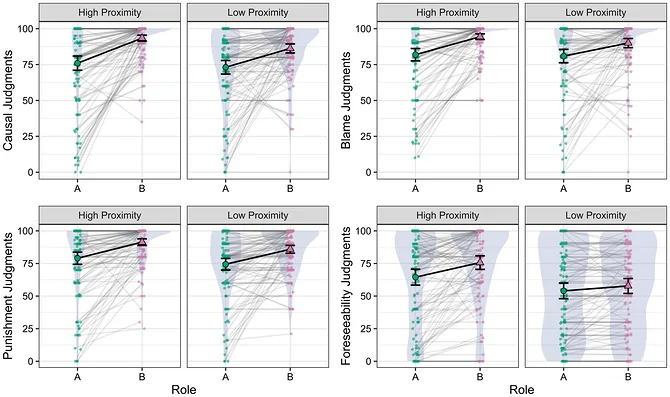
Moral and causal judgments for each agent in Study 2. Means and 95% CIs are indicated in black. Jitters show individual participant responses. Results for individual vignettes can be found in the Supplementary Appendix Section B.

We also found a significant main effect of proximity for all measures when responses are averaged across all three vignettes (see Fig. 3). We replicated the finding that participants judged both agents to be more of a cause, *F* (1, 250) = 4.83, *p* = .029, *η_p_* ^2^ = 0.02, and deserved more severe punishment, *F* (1, 250) = 5.38, *p* = .021, *η_p_* ^2^ = 0.02, in the high proximity condition when they more directly caused the outcome. However, we found no significant effect of proximity on judgments of blame, *F* (1, 250) = 1.40, *p* = .238, *η_p_* ^2^ = 0.006.

Participants in the high proximity condition also judged the outcome to be more foreseeable to both agents, *F* (1, 250) = 15.27, *p < *.001, *η_p_* ^2^ = 0.06. In other words, we found a similar pattern of results as in Cheung et al. (2024). Further, within each proximity condition, we found the same pattern of results for cause, blame, and punishment judgments as in Study 1 (all *p < *.001; see Table B1 in the Supplementary Appendix for mean scores).

The interaction between proximity and role was not significant for judgments except foreseeability, *F* (1, 250) = 11.21, *p < *.001, *η_p_* ^2^ = 0.04 (cause: *F* (1, 250) = 2.71, *p* = .101, *η_p_* ^2^ = 0.01; blame: *F* (1, 250) = 2.37, *p* = .125, *η_p_* ^2^ = 0.01; punishment: *F* (1, 250) = 0.23, *p* = .634, *η_p_* ^2^ *<* 0.001). In the low proximity condition, we found no significant difference in foreseeability judgments between A and B, *t*(250) = 2.27, *p* = .109; however, in the high proximity condition, participants judged the outcome to be more foreseeable to B, *t*(250) = 7.07, *p < *.001.

#### 3.2.2 Judgments of the victim

In the high proximity condition, while the victim (V) is the agent who is closest in time to the outcome in this causal chain of events, participants judged V almost completely not a cause (*M* = 2.0, 95% CI [0.8, 3.2]) nor blameworthy (*M* = 1.9, 95% CI [0.5, 3.8]).

### 3.3 Discussion

Overall, the presence of a subsequent contributing cause mitigated judgments for both A and B: *both agents* were more of a cause and more deserving of punishment when they *more directly* caused the outcome. This is consistent with previous findings that each action is more morally permissible in a longer chain of events, because they are each considered to be less likely and distal agents are less able to foresee the outcome (Engelmann & Waldmann, 2022). Further, participants still judged B more harshly compared to A overall. This suggests that while proximity to the final outcome does play a role in participants’ judgments, the relative proximity between A and B may also be a reason why B is considered more responsible.

Our results are in line with Sloman et al. (2009), who argue that intermediate causes are salient alternatives that attenuate the causal attribution of the effect to the distal cause. This is because the probability of the outcome occurring decreases as the length of the causal chain increases: there is an element of chance causing the outcome at any stage of the causal chain, which dilutes the causal attribution of agents (Sloman et al., 2009).

Cheung et al. (2024) found that when proximity to the outcome was reduced for both agents, participants judged A and B to be equally causal and deserving of punishment for the outcome. We replicated this finding only in the vignette we previously used in that study (Vignette 1), but not in the two new vignettes (Vignettes 2 and 3), where participants continued to judge B more severely than A. While this study was not sufficiently powered to detect differences between vignettes, it may still be worth discussing possible reasons for why the effect was stronger in Vignette 1. One explanation is the idiosyncrasies between vignettes: in Vignette 1, B was a “hired gun” whose extrinsic motivation (e.g., monetary gain) may have mitigated his responsibility. In Vignettes 2 and 3, B was a friend or acquaintance of A's, and his willingness to comply without clear incentives may have been perceived as reflecting a deeper moral failing. Inferred bad moral character and bad motives have been found to increase judgments of blame (Nadelhoffer, Knobe, & Nichols, 2008; for a review, see Carlson, Bigman, Gray, Ferguson, & Crockett, 2022), especially when information is limited, such as in short vignettes typically used in psychology experiments (Alicke & Rose, 2012; Cheung & Lagnado, 2023; Cheung, O'Neill, de Lanerolle, & Lagnado, 2026).

Further, both agents were highly blameworthy regardless of proximity to the outcome. This is supported by previous findings that people tend to blame others for having bad intentions or desires even if they did not directly cause a harmful outcome (Young & Saxe, 2011; Young & Tsoi, 2013). Both agents were judged to be less responsible when participants thought they did not foresee the outcome at the time they acted. This is consistent with previous research (Engelmann & Waldmann, 2022; Fincham & Shultz, 1981; Lagnado & Channon, 2008; Margoni & Brown, 2023) showing that foreseeability mitigates responsibility judgments.

One question that follows is whether agents would also be considered less responsible if they did not *intend* the final outcome to occur—as unintended outcomes are usually less foreseeable than intended ones (Kovacevic, Bonalumi, & Heintz, 2024). In our vignettes, the agents’ intentions were ambiguous: we only mentioned that A instructed B to *attack* V, and it is, therefore, unclear whether either agent had intended for V to die. It is possible that inferences about both agents’ intentions would influence moral judgments. We, therefore, aimed to further explore this in Study 3.

## 4 Study 3

In Study 3, we explored the interaction between intent and role by making intent unambiguous and directly manipulating the intent of each agent. Participants read the same causal chain scenario as Study 1—A instructs B to attack V, which directly leads to her death. However, we varied whether each defendant intended for the final outcome to occur (i.e., the victim's death). We explored (1) whether people would judge the agents differently depending on their intent, and (2) whether the causal chain between A and the outcome would weaken when B acts in a way that is not intended by A (and, therefore, the outcome was less foreseeable to A).

This study also investigated how participants represented the events in causal models. The first purpose of this was a manipulation check, to see whether participants accurately interpreted the causal structure of the scenario to be a causal chain. The second purpose was to explore whether differences in how people represented the events were associated with differences in how they attribute responsibility to the two agents.

### 4.1 Method

#### 4.1.1 Participants

The study was approved by the UCL Psychology Ethics Committee (EP/2018/005), and we obtained informed consent from all participants. We conducted an a priori power analysis for a mixed ANOVA. A pilot study revealed an effect size of *f* = 0.27 for the significant interaction between A's intent (between‐subjects) and role (within‐subjects), and a non‐significant interaction between B's intent (between‐subjects) and role (*f* = 0.14). Given budget constraints, it was not possible to power the study sufficiently to detect the latter. Thus, we used a more conservative effect size estimate to detect the interaction between A's intent and role. To achieve 80% power for detecting an effect size of *f* = 0.20 at an alpha‐level of 0.05, 136 participants were required.

We recruited 145 UK participants from Prolific with the same exclusion criteria as the previous studies (paid £2.90 for the 19‐min study). We excluded nine participants (one for failing the attention check, which asked what the scenario was about, and eight for not answering all questions). The final sample size was *N* = 136 (*M*_age_ = 38.7 years, *SD*_age_  = 13.2 years, 65 male, 71 female).

At the end of the experiment, we gave participants an additional comprehension check where they were asked to indicate which of the following statements corresponded to A and B's intentions as seen in the text: “Both A and B had intended to kill,” “Only A had intended to kill,” “Only B had intended to kill,” and “Nobody intended to kill.” A total of 99 participants passed the comprehension check, whereas 37 failed. We retained all participants in the final sample, as excluding those who failed the comprehension check showed the same pattern of results, and the comprehension failures are more likely to introduce noise than systematic bias.

#### 4.1.2 Design and materials

This study used a 2 (between‐subjects; A's Intent) *×* 2 (between‐subjects; B's Intent) *×* 2 (within‐subjects; role) design. Note that “intent” refers to intending the final outcome (i.e., killing V), not just intending the act (i.e., attacking V). In other words, “High Intent” refers to intending to kill V, whereas “Low Intent” refers to merely wanting to injure V. We showed a modified version of “Killing” with four different variations of intent.

- Both high intent: Both A and B intended to kill V *(A instructed B to kill V, B executed the killing)*;
- Both low intent: Both A and B did not intend to kill V *(A instructed B to* “*rough up” V, B intended to* “*rough up” but accidentally killed V)*;
- A high intent, B low intent: A intended to kill V but B did not *(A instructed B to kill V, B intended to* “*rough up” but accidentally killed V)*;
- B high intent, A low intent: A did not intend to kill, but B intended to kill V *(A instructed B to* “*rough up” V, B intended to kill V and executed the killing)*.

In all versions, the outcome is the same: V dies as a result of B's actions, which were brought about by A. For the within‐subjects measure (role), participants gave moral judgments for both defendants as in the previous studies.

#### 4.1.3 Procedure

First, participants were instructed on how to use Loopy (https://ncase.me/loopy/), an online web tool used to create causal models by drawing nodes and arrows. They engaged in two practice tasks where they constructed causal models from simple scenarios. Then, participants saw the experimental vignette, and they were asked to first represent the scenario using Loopy. Then, they gave causal and moral judgments as in the previous studies.

Finally, we asked participants to write a few lines to explain their judgments for the two agents in two separate questions.

#### 4.1.4 Data analysis

For the main analysis, we conducted 2 (between‐subjects; A's Intent) *×* 2 (between‐subjects; B's Intent) *×* 2 (within‐subjects; Role) mixed ANOVAs for each measure (cause, blame, and punishment) separately using afex (Singmann et al., 2022) and pairwise comparisons using emmeans (Lenth, 2022) with Tukey‐adjusted *p*‐values. To analyze the causal models constructed by participants, the first author (blind to the experimental conditions) categorized the causal structure of the models in four categories: A→B→V (causal chain), A→B→V, A→V (causal chain and conjunctive), and A→V→B (no causal chain, conjunctive), and as N/A (other or incorrect). Responses were coded as incorrect if they did not constitute a causal model (e.g., no nodes or no arrows).

We used participants’ responses to the open‐ended question explaining their judgments as a manipulation check. The first author (while blind to the experimental conditions) went through the responses and, whenever intentionality was mentioned for any agent, coded the response as indicating either high or low intent for each agent. For example, words such as “only wanted to hurt the victim,” “wanted her injured” were classified as having low intent, and words such as “deliberately,” “told…to kill” or “willed her death” were classified as high intent.

### 4.2 Results

#### 4.2.1 Manipulation check

While not all participants explicitly mentioned intent in the open response question, of those who did, most correctly identified the intent of the agents depending on the condition they were in (see Table 1). Additionally, participants tended to mention A's intent more than B's intent on average as part of their reason for mitigating or exacerbating blame for A. Conversely, they mostly mentioned B's physical actions in their reasoning for their judgments of B, rather than B's intent. The full results are available on OSF.

**Table 1:** Number of participants whose open responses specified high or low intent for each agent across conditions

Condition	High	Intent	Low	Intent	High	Intent	Low intent
	(A)		(A)		(B)		(B)
Both high intent	24		0		2		1
Both low intent	1		18		0		7
A high intent, B low intent	16		1		0		3
A low intent, B high intent	3						0

#### 4.2.2 Effect of role and intent

As shown in Fig. 4, overall, participants again judged B to be more of a cause than A, *F* (1, 132) = 28.87, *p < *.001, *η_p_* ^2^ = 0.18, more blameworthy than A, *F* (1, 132) = 9.56, *p* = .002, *η_p_* ^2^ = 0.07, and more deserving of severe punishment than A, *F* (1, 132) = 17.03, *p < *.001, *η_p_* ^2^ = 0.11 (see Table C1 in the Supplementary Appendix for mean scores).

**Fig. 4:**
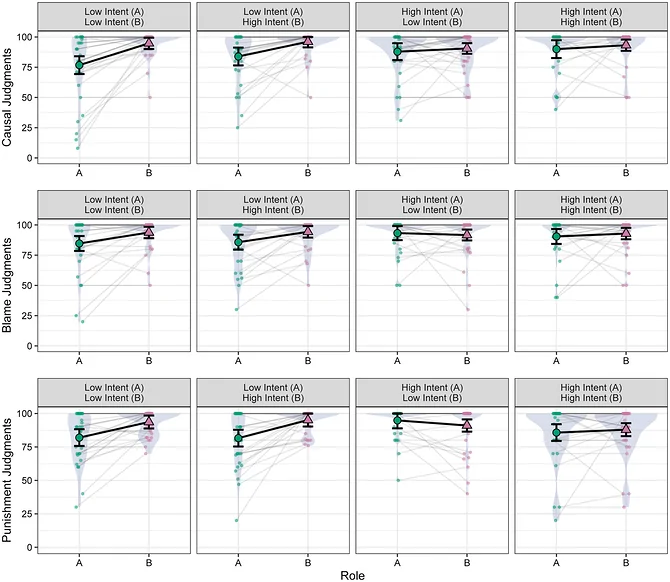
Moral and causal judgments for each agent at different levels of intent in Study 3. Means and 95% CIs are indicated in black. Jitters show individual participant responses.

We found a significant interaction between A's intent and role for judgments of cause, *F* (1, 132) = 13.10, *p < *.001, *η_p_* ^2^ = 0.09, blame, *F* (1, 132) = 7.98, *p* = .005, *η_p_* ^2^ = 0.06, and punishment, *F* (1, 132) = 22.09, *p < *.001, *η_p_* ^2^ = 0.14. In Fig. 4, the two panels on the left show how participants judged A and B differently when A had low intent: they judged A less harshly than B (all *p < *.001; see Supplementary Appendix for full results). When A had high intent, this difference between agents was not significant (all *p ≥ *.592). This is consistent with the results of the manipulation check, which also showed that participants tended to mention A's intent more. We found no evidence that B's intent influenced moral judgments between the two agents (cause: *F* (1, 132) = 0.61, *p* = .436, *η_p_* ^2^ = 0.005; blame: *F* (1, 132) = 0.33, *p* = .568, *η_p_* ^2^ = 0.003; punishment: *F* (1, 132) = 1.88, *p* = .173, *η_p_* ^2^ = 0.01).

Although some participants attributed maximal cause for both agents, the violin plots show that many more participants attributed maximal scores to B than for A. McNemar's tests revealed that when A had low intent, significantly more participants gave the maximal score to B than A for judgments of cause (75.8% vs. 33.3%, *χ* ^2^(1) = 22.8*, p < *.001), blame (72.7% vs. 45.5%, *χ*2(1) = 12.0*, p < *.001), and punishment (66.7% vs. 34.8%, *χ*2(1) = 16.0*, p < *.001). We found no evidence for this difference when A had high intent.

#### 4.2.3 Exploratory analysis: Effect of perceived causal structure

As an exploratory analysis and partially as a manipulation check, we investigated whether participants identified and correctly represented the events as a causal chain (across all conditions). One participant's response was excluded here due to a technical error with the Loopy link provided. As seen in Table 2, the majority of participants (72%; *N* = 98) represented the events as a causal chain (A→B→V). Of those participants, 67% represented the events using only the causal chain, and 33% also included an arrow between A and V (A→B→V, A→V), suggesting that they considered A's actions to be a direct (and also indirect) cause of the outcome.

**Table 2:** Most participants represented the scenario as a causal chain

Type of causal structure	Frequency
A→B→V	66 (49%)
A→B→V, A→V	32 (24%)
A→V→B	18 (13%)
Other or incorrect	20 (15%)

*Note*. Incorrect responses are those that do not constitute a causal model (e.g., no arrows). There is no evidence that participants’ causal attributions for A were impacted by how they perceived the causal structure of the scenario.

We conducted a mixed ANOVA to test the effect of causal structure, A's intent, B's intent, and role on participants’ causal judgments. We found no significant interaction between the type of causal structure participants drew and how much cause they attributed to each agent, *F* (3, 120) = 0.90, *p* = .444, *η_p_* ^2^ = 0.02, and no significant interaction when taking each agent's intent into account (Causal Structure *×* A's Intent *×* Role: *F* (3, 120) = 1.38, *p* = .251, *η_p_* ^2^ = 0.03; Causal Structure *×* B's Intent *×* Role: *F* (3, 120) = 0.79, *p* = .499, *η_p_* ^2^ = 0.02; four‐way: *F* (3, 120) = 1.35, *p* = .263, *η_p_* ^2^ = 0.03).

### 4.3 Discussion

We again showed that the executor (B) was generally more responsible, blameworthy, and deserving of punishment than the instigator (A). We also found an interaction between role and intent, such that participants gave more lenient judgments for the *instigator* when he did not intend the outcome—regardless of the executor's intentions. We found no evidence that the executor's intent impacted causal and moral judgments. Note, however, that the analysis was not sufficiently powered to detect the small effect of the interaction between B's intent and role.

These results are consistent with the doctrine of *novus actus interveniens* in criminal law, where a voluntary intervening cause only breaks the chain of causation when the outcome was not reasonably foreseeable to the distal agent. When A intended for V to die, then both the outcome and B's actions were reasonably foreseeable to him. Conversely, when A only intended for B to harm V and not to kill her, then both V's death and B's subsequent killing of V (regardless of whether it was intentional) could be deemed less foreseeable, making A less responsible. Put simply, this lack of intent for the outcome weakened the causal chain between A and the outcome. This is consistent with the findings of previous studies on how extending the causal chain can reduce causal judgments of an agent because their actions are perceived to be less reasonably foreseeable (Engelmann & Waldmann, 2022). In addition, our finding that A is judged more harshly for intentional actions is consistent with the legal realist view that intentional wrongfulness lengthens the reach of legal cause for the distal agent (Knobe & Shapiro, 2021). According to this view, moral wrongness of the action is taken into account when assessing proximate causation, and intentional wrongfulness is regarded as more morally wrong than unintentional actions.

## 5 Study 4

In Study 4, we build upon the existing studies and explore the interaction between intent, proximity, and role. We showed participants one of three vignettes based on those used in Study 2 with the added intentionality manipulation used in Study 3. With a larger sample size, the study was also sufficiently powered to detect the interaction between B's intent and role.

We hypothesized that we would replicate the main findings of previous studies, namely that (1) people will attribute more causality, blameworthiness, foreseeability, and punishment to the executor compared to the instigator; (2) both agents would receive lower causal and moral judgments in the low proximity condition; and (3) participants would judge A less harshly (compared to B) when A's intent is low (vs. when A's intent is high).

Further, the aim of this experiment was to investigate the interaction between A's intent, B's intent, proximity, and role on participants’ causal and moral judgments. For instance, is the effect of A's intent (B's intent) larger in the high proximity condition than in the low proximity condition? Do participants make different judgments between the agents when A's intent (B's intent) is low, more so in the high proximity condition than in the low proximity condition? Lastly, we also asked participants questions about counterfactual alternatives, and explored their evaluations of how likely the outcome would have occurred if each agent had been absent or acted differently (i.e., how necessary each agent was), and the relationship between these responses and their moral and causal judgments.

### 5.1 Method

#### 5.1.1 Participants

We preregistered the study at https://osf.io/dy5vq/. The study was approved by the UCL Psychology Ethics Committee (#0497), and we obtained informed consent from all participants. We conducted an a priori power analysis for a mixed ANOVA based on the results of previous studies.

We recruited 400 participants, which achieves over 95% power for detecting an effect size of *f* = 0.14 for a between‐within interaction with 8 *×* 2 conditions at an alpha‐level of 0.05. All participants were from the UK and recruited from Prolific with the same exclusion criteria as the previous studies (paid £1.05). We excluded 15 participants for failing one or more comprehension check questions, and one participant who revoked their consent for participating in the study. The final sample size was, therefore, *N* = 384 (*M*_age_ = 45.1 years, *SD*_age_ = 13.5 years, 193 male, 191 female).

#### 5.1.2 Design, materials, and procedure

This study used a 2 (between‐subjects; A's Intent) *×* 2 (between‐subjects; B's Intent) *×* 2 (between‐subjects; Proximity) *×* 2 (within‐subjects; Role) design. Each participant was randomly assigned to one of three vignettes, but note that the study does not have sufficient power to detect effects in each individual vignette. We used three vignettes based on materials in Studies 2 and 3 (see OSF for full materials). We used the same judgment measures as in previous studies (cause, blame, foreseeability, and punishment). We added the following counterfactual questions for each agent: “If it weren't for [agent name], how likely would the outcome still have happened?” on scales of 0 (extremely unlikely) to 100 (extremely likely).

Participants also answered the following counterfactual questions. In the high proximity condition, we asked: (1) “Imagine that A asked B to attack V, but B refused or was unavailable. How likely would A have asked somebody else?”, (2) “Imagine that A asked B to attack V, but B refused or was unavailable. How likely would A have done it himself?”, and (3) “Imagine that A never reached out to B. How likely would B have caused someone's death in a different situation?”. In the low proximity condition, we asked an additional question: (4) “Imagine that V did not [experience the additional cause]. How likely would the outcome still have happened?”. All questions were answered on scales of 0 (extremely unlikely) to 100 (extremely likely).

As a manipulation check, we asked participants (1) whether each defendant intended to cause the victim's death (yes/no), and (2) whether there was, aside from A's instruction and B's attack, any additional event that contributed to the victim's death (yes/no).

#### 5.1.3 Data analysis

For the main analysis, we conducted 2 (between‐subjects; A's Intent) *×* 2 (between‐subjects; B's Intent) *×* 2 (between‐subjects; Proximity) *×* 2 (within‐subjects; Role) mixed ANOVAs using afex (Singmann et al., 2022) and pairwise comparisons using emmeans (Lenth, 2022) with Tukey‐adjusted *p*‐values. We also included Vignette as a factor to account for variability, but due to not having sufficient power, we will not interpret main effects and interactions involving Vignette.

In an exploratory analysis, we used multiple regression to test whether participants’ responses to five counterfactual questions predict causal and blame attributions for each agent, controlling for all other predictors simultaneously. We report Holm‐corrected *p*‐values for each outcome variable.

### 5.2 Results

#### 5.2.1 Manipulation check

Most participants passed the manipulation check.^3^ For the proximity manipulation, we asked participants whether there was an additional event that contributed to the outcome besides A and B's involvement. In the high proximity condition, 82.6% of participants answered correctly, and in the low proximity condition, 95.5% answered correctly. For the intentionality conditions, we asked participants whether each agent intended for the victim to die. Between 76.97.0% and 87.9% of participants answered correctly. We included all participants in the main analysis (as preregistered); excluding those who failed the comprehension check showed the same pattern of results.

#### 5.2.2 Role, intent, and proximity

As predicted, we replicated the finding that participants judged B more harshly than A overall (cause: *F* (1, 360) = 231.57, *p < *.001, *η_p_* ^2^ = 0.39; blame: *F* (1, 360) = 113.26, *p < *.001, *η_p_* ^2^ = 0.24; foreseeability: *F* (1, 360) = 118.64, *p < *.001, *η_p_* ^2^ = 0.25; punishment: *F*(1,360) = 174.77, *p* < .001,*η_p_* ^2^ = 0.33).

Fig. 5 shows that participants judged agents slightly more harshly in the high proximity condition in almost all panels. However, we did not find a significant main effect of proximity on most measures (cause: *F* (1, 360) = 0.42, *p* = .517, *η_p_* ^2^ *<* 0.001, blame:

**Fig. 5:**
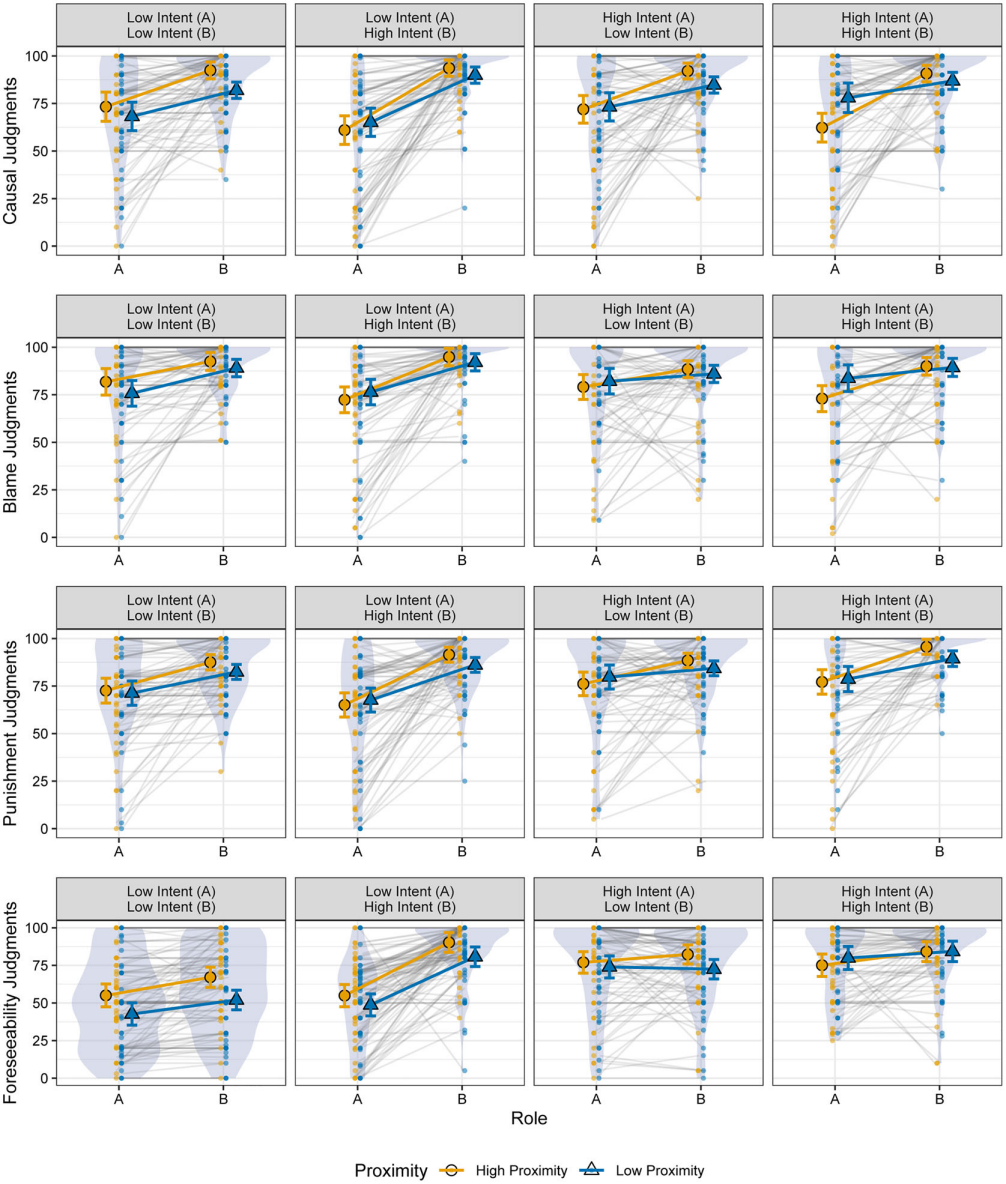
Moral and causal judgments for each agent in Study 4. Error bars show 95% CIs. Jitters show individual participant responses. Results for individual vignettes can be found in the Supplementary Appendix Section D.

*F* (1, 360) = 0.03, *p* = .860, *η_p_* ^2^ = 0.001, punishment: *F* (1, 360) = 1.32, *p* = .251, *η_p_* ^2^ = 0.004) except foreseeability (*F* (1, 360) = 8.37, *p* = .004, *η_p_* ^2^ = 0.023).

We replicated the significant interaction between A's intent and role. When A had high intent, the difference between the agents became significantly smaller in all judgment measures (cause: *F* (1, 360) = 4.10, *p* = .044, *η_p_* ^2^ = 0.011, blame: *F* (1, 360) = 8.17, *p* = .004, *η_p_* ^2^ = 0.022; punishment: *F* (1, 360) = 7.93, *p* = .005, *η_p_* ^2^ = 0.022, foreseeability: *F* (1, 360) = 54.13, *p < *.001, *η_p_* ^2^ = 0.131). Consistent with the literature, when A's intent is high, participants gave much higher foreseeability judgments for A compared to B (from *M* = 50.3 to *M* = 76.5, *t*(360) = 9.81*, p < *.001). This interaction effect was mostly driven by increases in judgment scores for A for punishment judgments (A: *t*(360) = 3.83, *p < *.001 vs. B: *t*(360) = 1.80, *p* = .273). However, neither simple effects were significant for causal judgments (both *p ≥ *.355) and blame judgments (both *p ≥ *.116).

We also found a significant interaction between B's intent and role. When B had high intent, the difference between the agents became significantly larger in all judgment measures: (cause: *F* (1, 360) = 8.32, *p* = .004, *η_p_* ^2^ = 0.023; blame: *F* (1, 360) = 6.61, *p* = .011, *η_p_* ^2^ = 0.018; punishment: *F* (1, 360) = 12.41, *p < *.001, *η_p_* ^2^ = 0.033; foreseeability: *F* (1, 360) = 32.63, *p < *.001, *η_p_* ^2^ = 0.083). For cause and blame judgments, neither simple effects in each role level were significant (all *p ≥ *.240), suggesting that the difference was driven by judgments in both directions. For punishment and foreseeability judgments, the interaction was driven by increased judgments for B (*t*(360) = 3.50, *p* = .003 and *t*(360) = 6.95, *p < *.001, respectively), while judgments for A were not significantly different (both *p ≥ *.613).

We did not find a significant interaction between A's intent and proximity for most judgments (all *p* ≥ .142 = ) except foreseeability, *F* (1, 360) = 4.03, *p* = .046, *η_p_* ^2^ = 0.011. The effect of A's intent on judgments for both agents was larger in the low proximity condition. We also did not find any significant three‐way interaction effects between A's intent, role, and proximity, and B's intent, role, and proximity (both *p ≥ * .098).

#### 5.2.3 Exploratory analysis: Counterfactual questions

First, we analyzed participants’ responses to the counterfactual question: “If it weren't for [agent name], how likely would the outcome still have happened?”. Participants judged the outcome (the victim's death) to be significantly less likely to occur without A (*M* = 14.8%, 95% CI [12.6, 17.1]) compared to without B (*M* = 22.6%, 95% CI [19.6, 25.5]), *t*(383) = −5.04*, p < *.001, *d* = −0.26. This suggests that A was perceived as more necessary for the outcome, despite the earlier finding that participants tended to judge B as more of a cause and more blameworthy overall.

Next, we investigated the relationship between counterfactual responses and participants’ judgments of cause and blame for each agent. First, we found that the more participants thought the outcome would still occur without A, the less cause and blame they attributed to him (cause: *b* = −0.18, *t*(378) = −2.69, *p* = .029; blame: *b* = −0.21, *t*(378) = −3.40, *p* = .003). We found no significant effect of counterfactual responses for A on judgments for B (both *p* = 1.00). Further, the more participants thought the outcome would still occur without B, the more cause they assigned to A (*b* = 0.14, *t*(378) = 2.70, *p* = .029; relationship with blame was not significant, *p* = .085). Conversely, counterfactual responses for B did not significantly predict judgments for B (both *p ≥ *.633).

Overall, in three additional counterfactual questions about the specific actions of A and B, participants reported that it was quite likely for A to have hired someone else to commit the crime if B had not been available (*M* = 68.6%, 95% CI [66.0, 71.2]). They also reported that it was less likely that A would have carried out the harm personally (*M* = 43.3%, 95% CI [40.5, 46.2]), or that B would have committed harm without A's involvement (*M* = 40.7%, 95% CI [37.8, 43.7]). This is consistent with the finding from the previous counterfactual questions that B was judged to be less of a necessary cause than A.

We also investigated the relationship between these counterfactual responses and participants’ judgments of cause and blame for each agent. As shown in Fig. 6, counterfactual responses about what A would do in B's absence significantly predicted participants’ judgments of cause and blame. Believing A would have hired someone else (if B had been unavailable) predicted higher judgments for A (cause: *b* = 0.32, *t*(378) = 5.44, *p < *.001; blame: *b* = 0.27, *t*(378) = 5.13, *p < *.001), as did believing A would have conducted the act himself personally instead (cause: *b* = 0.21, *t*(378) = 4.23, *p < *.001; blame: *b* = 0.17, *t*(378) = 3.78, *p* = .001). In contrast, believing B would have committed harm without A's involvement did not predict judgments for A (both *p ≥ *.237). Notably, responses to the counterfactual questions did not significantly predict causal or blame judgments for B after correcting for multiple comparisons (all *p ≥ *.086).

**Fig. 6:**
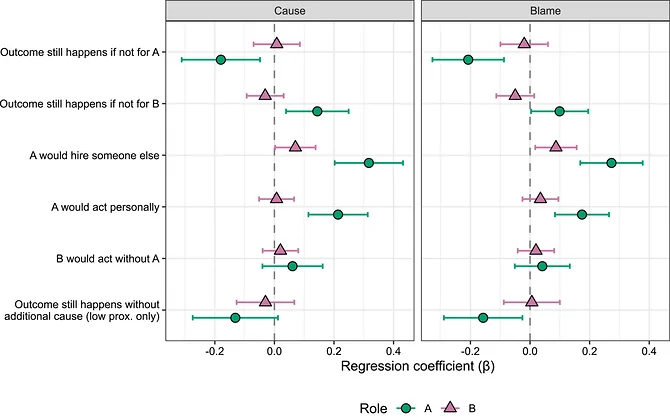
Responses to counterfactual questions revealed that participants judged A to be more of a necessary cause than B, and also significantly predicted judgments of cause and blame for A, but not for B. Points show unstandardized regression coefficients (*b*); error bars show 95% CI.

In the low proximity condition, we asked participants how likely the outcome would still have happened if V did not experience the additional cause (i.e., the cause independent of A and B's actions). High reported likelihoods that the outcome would still have happened did not significantly predict judgments for A nor B (all *p ≥ *.059). Overall, these results suggest that although participants consistently judged B as more causal and blameworthy than A, they also perceived A as more necessary for the outcome. Further, the more participants viewed B as replaceable or unnecessary, the more cause and blame they attributed to A. By contrast, participants’ counterfactual responses about B's role had a much smaller or nonsignificant effect on judgments of either agent.

### 5.3 Discussion

Overall, we replicated most of the effects from the previous studies. Participants consistently assigned higher causality, blame, foreseeability, and punishment judgments to the executor (B) compared to the instigator (A). We also saw an effect of both A's intent and B's intent, whereby agents with high intent were generally judged more harshly However, we did not find the effect of proximity that we previously found in Study 2.

One reason why we did not find a significant effect of proximity may be because we explicitly manipulated the intentionality of both agents. When this information was given, participants may have focused more on the agents’ mental states instead of how directly they caused the outcome. This may be because proximity is very closely linked to foreseeability. Indeed, we found a main effect of proximity on foreseeability judgments: participants judged that the agents were more likely to have foreseen the outcome when they were more proximal to it. By manipulating intentionality, it is possible that we also manipulated perceived foreseeability, as intentionality itself also reveals information about foreseeability; intending an outcome to occur necessitates foreseeing the possibility of the outcome occurring. In other words, our intentionality manipulation may have overshadowed the pathway through which proximity typically influences moral judgments.

Interestingly, the exploratory analysis showed that participants considered A more of a robust cause: on average, participants reported that it was quite likely A would have hired someone else to commit the crime if B had been absent, and also considered B to be less necessary for the outcome. Despite this, participants still attributed more cause to B. We discuss this in more detail in the following section.

## 6 General discussion

In four studies, we explored how laypeople attribute causal responsibility in a causal chain where an agent (A) instructs an intermediate agent (B) to execute a harmful action resulting in the death of a victim (V). Study 1 showed that participants tended to judge the executor (B) to be more of a cause, more blameworthy, and more deserving of severe punishment than the instigator (A). Study 2 revealed the importance of proximity, because when the proximity to the harm for A and B decreases, attributions of causal responsibility and punishment were reduced for both agents, though higher ratings for B versus A persisted. Further, Studies 3 and 4 showed that higher intentionality largely drove harsher judgments for each agent. While A's high intent reduced blame for B, B's intent did not influence judgments for A.

### 6.1 Why do participants assign more cause to the executor rather than the instigator?

Across all our studies, participants attributed higher causal responsibility to the executor (B) than the instigator (A). This is a novel finding that poses a challenging test case for current psychological theories of causal judgment. It cannot be explained by a simple counterfactual account, which argues that X causes Y only if Y would not have occurred without X. In some sense, the instigator and executor are equally causal, as the specific outcome would not have occurred had either failed to act. However, in another sense, they are not equally counterfactually causal. That is because the executor could be replaced by someone else, rendering this particular person unnecessary to achieve the executor's goal. Participants’ responses to the counterfactual questions revealed that they may have agreed with this assessment, as they judged the outcome to be more likely without the executor than without the instigator. Even so, they surprisingly still attributed higher causal ratings overall for the executor. This must be explained by something other than counterfactual reasoning.

An explanation solely in terms of proximity also falls short. Proximity is open to various interpretations (e.g., temporal, spatial). If we restrict the definition to temporal proximity, then it seems insufficient to explain the consistent higher causal attribution to the executor over the instigator. In Study 2, the temporal proximity between executor and outcome was extended, and yet, the preference for executor over instigator persisted. Moreover, in Study 4, the effect of proximity to the outcome was likely overridden by the manipulation of the agents’ intentions. However, it is difficult to rule out temporal proximity entirely, because, by necessity, the executor acts after the instigator.

Therefore, rather than focusing on only *temporal* proximity, we may also consider *physical* proximity. One possible explanation, noted in the Introduction, is that participants rated the executor higher because of his direct contact (and the force he exerted) on the victim (Greene et al., 2009). This fits with psychological theories of causation that emphasize the role of force (Wolff & Thorstad, 2017) or process/mechanism (Walsh & Sloman, 2011). It also fits with the notion of how‐causation (Gerstenberg et al., 2021; Lewis, 1973), where people's causal judgments are sensitive to how modifications to the cause would lead to variations in the effect. For example, the executor is more of a how‐cause of the victim's death, because modifications in his actions—exactly how he attacks the victim—will change the exact nature of the victim's injuries more than modifications in the instructions given by the instigator. Future work could explore these possibilities in more detail.

Participants also considered the ultimate harm as more foreseeable for the executor compared to the instigator: the executor might still forgo the plan for many reasons (e.g., they might change their mind, lose their nerve, or get interrupted). In this sense, our studies of lay attitudes track the common law doctrine that finds greater causation and responsibility for more foreseeable harms. Further, people's causal judgments were strongly moderated by intentionality; in line with many theories of blame (Alicke, 2000; Malle & Knobe, 1997) and empirical findings (e.g., Ames & Fiske, 2013; Lagnado & Channon, 2008), agents were considered more causal and blameworthy when their actions were intentional. This was especially the case for the executor; when he both committed the physical act and intended for the victim to die, participants’ average judgments reached the ceiling in almost all conditions.

Together, our results suggest that lay causal attributions go beyond a “but‐for” test of causation and simple judgments of necessity. Participants were likely considering other features relevant to the agents’ intentions and behavior, such as moral norm violations of committing a harmful physical act (e.g., Knobe & Fraser, 2008).

This opens up several avenues for future research. First, to more directly test the relationship between participants’ imagined counterfactuals and moral judgments, future work could manipulate the counterfactuals that participants consider, such as using processing‐tracing techniques (Zhao, Richie, & Bhatia, 2022). For instance, we could ask participants to first identify relevant counterfactuals in a thought‐listing task and then direct them to imagine specific ones. Second, temporal and physical proximity could be disentangled: for example, how would participants attribute cause and blame in a scenario where A tells B to poison V's drink, causing V to die immediately? While B's action of adding poison causes V's death in a temporally proximal way, it may be considered less direct than a physical attack. Another example is one of omission, where the harm results because agents in a causal chain failed to intervene. Third, in our studies, we asked participants to give judgments on the extent to which each agent could have foreseen the outcome at the time of acting. Future work could also measure foreseeability in terms of the manner in which the outcome occurs in scenarios where this measure would be appropriate, for example, in cases where multiple agents are involved in carrying out a command. Lastly, though we used multiple vignettes in Studies 2 and 4, future work could include vignettes which systematically vary different aspects of the scenarios, for example, by manipulating the involved parties’ agency over their actions (e.g., how much choice the “hired gun” has).

## 7 Participants’ judgments are somewhat consistent with the law

Whereas the criminal law does not treat those who carry out a crime as inherently more culpable than those who solicit it, our studies suggest that the executor's more direct and proximate connection to the harm renders them more causal, blameworthy, and deserving of severe punishment to the folk. Even when we introduced a novel, additional intervening act taken by the victim (the low proximity condition), which attenuated the directness of B's actions and how much both agents could have foreseen the outcome, B was still judged more harshly.

This likely reflects an ability to toggle from foreseeability to alternative bases for responsibility when foreseeability is absent, but participants are motivated to blame. Where the executor's conduct is particularly morally reprehensible, participants may have looked past their judgment that the harm is unforeseeable, and instead relied on how the executor's behavior was voluntary, transactional, and a more direct, temporally close, and substantial factor in the harm. That is, higher foreseeability may be sufficient but not necessary to increase assessments of causation and blame. This finding helps to clarify the inconsistent reliance on foreseeability in actual criminal cases. Finally, while the analysis of intervening causes under legal causation should be distinct from the analysis of “but‐for” factual causation, participants might blur the two. For instance, they may consider that the executor had the last opportunity to prevent the harm.

By bridging the gap between psychological theories of causation and real‐world applications, our findings provide important insights into folk attribution of causal responsibility in cases involving multiple agents acting in concert. These results also reveal why judges in these complex cases might be toggling between different tests for when intervening causes should be superseding—they might be switching from foreseeability to intent or from foreseeability to proximity. Understanding these intuitions is, therefore, not only of theoretical interest: it may help explain, and perhaps even improve, how the legal system determines responsibility and punishment when wrongdoing is shared between individuals.

## Funding

This work was partially supported by the Institute for Humane Studies under grant number IHS01785 to V.C. V.C. was also supported by a Funds for Women Graduates Foundation Grant, which provided a graduate bursary to help with living expenses.

## Conflicts of interest

The authors declare no known conflicts of interest.

## Ethics approval statement

All studies were approved by the UCL Psychology Ethics Committee (EP/2018/005 and 0497), and we obtained informed consent from all participants.

## Supporting information



Supporting Information

## Data Availability

All data, materials, and analysis scripts are available at the OSF repository (https://osf.io/bg5xz/). Study 2 was preregistered at https://osf.io/xrqaw and Study 4 was preregistered at https://osf.io/dy5vq/. Studies 1 and 3 were not preregistered and previously reported in the nonarchival Proceedings of the Annual Meeting of the Cognitive Science Society (https://escholarship.org/uc/item/7zr9p3jv).

## Notes

1
https://www.justice.gov/usao‐ndtx/pr/jennifer‐faith‐pleads‐guilty‐murder‐hire‐husband‐s‐death

2
https://www.nbcnews.com/news/us‐news/darrin‐lopez‐jennifer‐faith‐love‐triangle‐murder‐army‐veteran‐rcna117945

3 In this section, we report a sample excluding the first 30 participants who took part in our study, as they did not see the proximity manipulation check due to a programming error. These participants were still included in the main analysis.
