# Supplementary material for: Attribution of Responsibility Between Agents in a Causal Chain
Source: Cogn Sci. 2026 Nov 10;50(11):e70280. doi: 10.1111/cogs.70280 (PMC13752550; doi:10.1111/cogs.70280)
Supplement: Supplementary file 1 [file COGS-50-e70280-s001.docx]

# Appendix A Supplementary Results for Study 1

100

Cause

Blame

Punishment

Free-Response Judgment Scores

75

50

25

0

A	B	A	B	A	B

Role

# Figure A1

*Free response judgments of cause, blame, and punishment ratings distributed to each agent in Study 1. Means and 95% CIs are indicated in black. Jitters show individual participant responses.*

In participants’ free allocation responses, they judged B to be more of a cause than A, *t*(89) = 5*.*35, *p < .*001, *d_rm_* = 0*.*62, more blameworthy than A, *t*(89) = 3*.*79, *p < .*001, *d_rm_* = 0*.*34, and deserving of more severe punishment than A, *t*(89) = 3*.*99, *p < .*001,

*d_rm_* = 0*.*35.

# Table B1

**Appendix B Supplementary Results for Study 2**

*Mean Scores and 95% Confidence Intervals for Cause, Blame, and Punishment by Role and Proximity Condition in Study 2*

|  | | **High Proximity** | **Low Proximity** |
| --- | --- | --- | --- |
| **Cause** | **A** | 75*.*8 [71.3, 80.3] | 73*.*3 [68.8, 77.9] |
|  | **B** | 93*.*5 [90.9, 96.1] | 86*.*2 [83.6, 88.8] |
| **Blame** | **A** | 81*.*7 [77.7, 85.8] | 81*.*1 [77.0, 85.2] |
|  | **B** | 94*.*4 [91.8, 97.1] | 90*.*0 [87.3, 92.7] |
| **Punishment** | **A** | 78*.*9 [75.0, 82.8] | 74*.*7 [70.7, 78.6] |
|  | **B** | 91*.*3 [88.6, 94.0] | 85*.*9 [83.2, 88.7] |
| **Foreseeability** | **A** | 64*.*2 [59.0, 69.3] | 54*.*3 [49.0, 59.5] |
|  | **B** | 75*.*5 [70.5, 80.6] | 58*.*0 [52.8, 63.1] |

**Vignette 1**

100	100

High Proximity

Low Proximity

High Proximity

Low Proximity

75	75

Causal Judgments

Blame Judgments

50	50

25	25

0

A	B	A	B

0

A	B	A	B

100	100

High Proximity

Low Proximity

High Proximity

Low Proximity

Punishment Judgments

Foreseeability Judgments

75	75

50	50

25	25

0

A	B	A	B

Role

0

A	B	A	B

Role

# Figure B1

*Moral and Causal Judgments for Vignette 1 in Study 2. Means and 95% CIs are indicated in black. Jitters show individual participant responses.*

**Vignette 2**

100	100

High Proximity

Low Proximity

High Proximity

Low Proximity

75	75

Causal Judgments

Blame Judgments

50	50

25	25

0

A	B	A	B

0

A	B	A	B

100	100

High Proximity

Low Proximity

High Proximity

Low Proximity

Punishment Judgments

Foreseeability Judgments

75	75

50	50

25	25

0

A	B	A	B

Role

0

A	B	A	B

Role

# Figure B2

*Moral and Causal Judgments for Vignette 2 in Study 2. Means and 95% CIs are indicated in black. Jitters show individual participant responses.*

**Vignette 3**

100	100

High Proximity

Low Proximity

High Proximity

Low Proximity

75	75

Causal Judgments

Blame Judgments

50	50

25	25

0

A	B	A	B

0

A	B	A	B

100	100

High Proximity

Low Proximity

High Proximity

Low Proximity

Punishment Judgments

Foreseeability Judgments

75	75

50	50

25	25

0

A	B	A	B

Role

0

A	B	A	B

Role

# Figure B3

*Moral and Causal Judgments for Vignette 3 in Study 2. Means and 95% CIs are indicated in black. Jitters show individual participant responses.*

# Appendix C Supplementary Results for Study 3

**Table C1**

*Means and 95% CIs for cause, blame, and punishment judgments across Role and levels of A’s Intent in Study 3*

|  | **Agent** | **High Intent (A)** | **Low Intent (A)** |
| --- | --- | --- | --- |
| **Cause** | **A** | 88.9 [83.8, 94.0] | 80.3 [75.1, 85.5] |
|  | **B** | 91.8 [88.7, 95.0] | 95.4 [92.1, 98.7] |
| **Blame** | **A** | 91.9 [87.6, 96.1] | 85.2 [80.8, 89.6] |
|  | **B** | 92.3 [89.0, 95.5] | 94.0 [90.7, 97.4] |
| **Punishment** | **A** | 90.3 [86.0, 94.6] | 81.8 [77.4, 86.2] |
|  | **B** | 89.5 [86.2, 92.8] | 94.4 [91.0, 97.8] |

Pairwise comparisons revealed that, when A had Low Intent, participants judged A to be less of a cause than B, *t*(132) = 6*.*27, *p < .*001. When A had high intent, difference between judgments for the two agents was not significant, *t*(132) = 1*.*26*, p* = *.*592. We found a similar pattern of results for blame judgments. Participants judged A to be significantly less blameworthy than B when A had Low Intent, *t*(132) = 4*.*13, *p < .*001.

When A had High Intent, judgments of blame did not significantly differ between A and B, *t*(132) = 0*.*19, *p* = *.*998. For punishment judgments, participants judged A to be less deserving of punishment than B when A had Low Intent, *t*(132) = 6*.*16, *p < .*001. When A had High Intent, judgments again did not significantly differ between A and B,

*t*(132) = 0*.*41, *p* = *.*977.

**Appendix D**

**Supplementary Results for Study 4**

**Vignette 1**

100

Low Intent (A) Low Intent (B)

Low Intent (A) High Intent (B)

High Intent (A) Low Intent (B)

High Intent (A) High Intent (B)

75

Causal Judgments

50

25

0

A	B	A	B	A	B	A	B

100

Low Intent (A) Low Intent (B)

Low Intent (A) High Intent (B)

High Intent (A) Low Intent (B)

High Intent (A) High Intent (B)

75

Blame Judgments

50

25

0

A	B	A	B	A	B	A	B

100

Low Intent (A) Low Intent (B)

Low Intent (A) High Intent (B)

High Intent (A) Low Intent (B)

High Intent (A) High Intent (B)

Punishment Judgments

75

50

25

0

A	B	A	B	A	B	A	B

100

High Intent (A) High Intent (B)

High Intent (A) Low Intent (B)

Low Intent (A) High Intent (B)

Low Intent (A) Low Intent (B)

Foreseeability Judgments

75

50

25

0

A	B	A	B	A	B	A	B

Role

# Figure D1

Proximity High Proximity Low Proximity

*Moral and Causal Judgments for Vignette 1 in Study 4. Error bars show 95% CIs. Jitters show individual participant responses.*

# Vignette 2

100

Low Intent (A) Low Intent (B)

Low Intent (A) High Intent (B)

High Intent (A) Low Intent (B)

High Intent (A) High Intent (B)

75

Causal Judgments

50

25

0

A	B	A	B	A	B	A	B

100

Low Intent (A) Low Intent (B)

Low Intent (A) High Intent (B)

High Intent (A) Low Intent (B)

High Intent (A) High Intent (B)

75

Blame Judgments

50

25

0

A	B	A	B	A	B	A	B

100

Low Intent (A) Low Intent (B)

Low Intent (A) High Intent (B)

High Intent (A) Low Intent (B)

High Intent (A) High Intent (B)

Punishment Judgments

75

50

25

0

A	B	A	B	A	B	A	B

100

Low Intent (A) Low Intent (B)

Low Intent (A) High Intent (B)

High Intent (A) Low Intent (B)

High Intent (A) High Intent (B)

Foreseeability Judgments

75

50

25

0

A	B	A	B	A	B	A	B

Role

# Figure D2

Proximity High Proximity Low Proximity

*Moral and Causal Judgments for Vignette 2 in Study 4. Error bars show 95% CIs. Jitters show individual participant responses.*

100

Low Intent (A) Low Intent (B)

Low Intent (A) High Intent (B)

High Intent (A) Low Intent (B)

High Intent (A) High Intent (B)

75

Causal Judgments

50

25

0

A	B	A	B	A	B	A	B

100

Low Intent (A) Low Intent (B)

High Intent (A) Low Intent (B)

High Intent (A) High Intent (B)

Low Intent (A) High Intent (B)

75

Blame Judgments

50

25

0

A	B	A	B	A	B	A	B

100

Low Intent (A) Low Intent (B)

Low Intent (A) High Intent (B)

High Intent (A) High Intent (B)

High Intent (A) Low Intent (B)

Punishment Judgments

75

50

25

0

A	B	A	B	A	B	A	B

100

Low Intent (A) Low Intent (B)

High Intent (A) High Intent (B)

High Intent (A) Low Intent (B)

Low Intent (A) High Intent (B)

Foreseeability Judgments

75

50

25

0

A	B	A	B	A	B	A	B

Role

# Figure D3

Proximity High Proximity Low Proximity

*Moral and Causal Judgments for Vignette 3 in Study 4. Error bars show 95% CIs. Jitters show individual participant responses.*
